# Contrast-enhanced MRI findings of the knee in healthy children; establishing normal values

**DOI:** 10.1007/s00330-017-5067-6

**Published:** 2017-10-06

**Authors:** Robert Hemke, J. Merlijn van den Berg, Charlotte M. Nusman, E. Charlotte van Gulik, Anouk M. Barendregt, Dieneke Schonenberg-Meinema, Koert M. Dolman, Taco W. Kuijpers, Mario Maas

**Affiliations:** 1 Department of Radiology and Nuclear Medicine, Academic Medical Center, University of Amsterdam, Meibergdreef 9, 1105AZ Amsterdam, The Netherlands; 2 Department of Pediatric Hematology, Immunology, Rheumatology and Infectious Diseases, Emma Children’s Hospital AMC, University of Amsterdam, Meibergdreef 9, Amsterdam, The Netherlands; 3 Department of Pediatrics, Onze Lieve Vrouwe Gasthuis (OLVG), Jan Tooropstraat 164, Amsterdam, The Netherlands; 4 Department of Pediatric Rheumatology, Reade, Dr. Jan van Breemenstraat 2, Amsterdam, The Netherlands

**Keywords:** Magnetic resonance imaging, Synovitis, Normal values, Knee joint, Children

## Abstract

**Objectives:**

To define normative standards for the knee in healthy children using contrast-enhanced MRI, focusing on normal synovial membrane thickness. Secondly, presence of joint fluid and bone marrow oedema was evaluated.

**Methods:**

For this study, children without disorders potentially resulting in (accompanying) arthritis were included. Patients underwent clinical assessments, followed by contrast-enhanced MRI. MRI features were evaluated in consensus using the Juvenile Arthritis MRI Scoring (JAMRIS) system. Additionally, the presence of joint fluid was evaluated. No cartilage lesions or bone abnormalities were observed.

**Results:**

We included 57 healthy children. The overall mean thickness of the normal synovial membrane was 0.4 mm (min–max; 0.0–1.8mm). The synovium was thickest around the cruciate ligaments and retropatellar and suprapatellar regions. The mean overall diameter of the largest pocket of joint fluid was 2.8 mm (min–max; 0.9–8.0mm). Bone marrow changes were observed in three children (all in the apex patellae).

**Conclusions:**

The normal synovial membrane was maximally 1.8 mm thick, indicating that the JAMRIS cut-off value of 2 mm can be considered a valid measure for evaluating synovial hypertrophy. Some joint fluid and bone marrow changes suggestive of bone marrow oedema in the apex patellae can be seen in healthy children.

***Key Points*:**

• *Knowledge on the normal synovial appearance using contrast-enhanced MR is lacking*.

• *In healthy children, normal synovial membrane is maximally 1.8 mm thick*.

• *Normal synovium is thickest around the cruciate ligaments, retropatellar and suprapatellar*.

• *Bone marrow oedema in the apex patellae is seen in healthy children*.

## Introduction

Magnetic resonance imaging (MRI) is considered to be the reference standard for the assessment of disease status in patients with juvenile idiopathic arthritis (JIA) [1, 2]. Although contrast-enhanced MRI is the preferred imaging modality for the evaluation of synovial thickening and destructive changes in JIA [3–5], its use in the knee as the most commonly affected joint in JIA is severely hampered by the lack of knowledge on the normal appearance of this joint in healthy children [6, 7]. Differentiating physiological from pathological appearances of the growing joint can, therefore, be very challenging. For instance, bony depressions and signal changes suggestive of bone marrow oedema are commonly seen in wrists of healthy children [8, 9].

Consequently, MRI is underutilised in both clinical practice and research. Therefore, the objective of this study was to define normative standards for the knee in healthy children using contrast-enhanced MRI, focusing on the normal thickness of the contrast-enhanced synovial membrane. Moreover, the presence of joint fluid, bone marrow changes suggestive of bone marrow oedema, cartilage lesions and bone abnormalities suggestive of bone erosions were evaluated.

## Materials and methods

### Patients

Children who had MRI evaluation of the knee and who had (1) clinically active arthritis (suspected JIA, new-onset JIA or remitting/relapsing JIA) or JIA patients who had (2) clinically inactive disease and a history of clinical evident arthritis in at least one knee, were prospectively included between December 2008 and July 2016. Children visited one of three collaborating outpatient clinics, i.e. a tertiary paediatric rheumatology centre (Academic Medical Centre, Amsterdam, The Netherlands) or one of two non-academic paediatric rheumatology centres (Reade and Onze Lieve Vrouwe Gasthuis, both Amsterdam, The Netherlands). At the time of presentation all patients underwent clinical and laboratory assessments, followed by contrast-enhanced MRI of the knee.

For the purpose of this study, we included children without juvenile idiopathic arthritis or any inflammatory or orthopaedic disorder potentially resulting in accompanying arthritis.

Inclusion criteria were: (1) children with passing knee complaints without a known cause, (2) patients with joint complaints based on hypermobility, or (3) complaints based on a functional disorder/chronic pain syndrome.

For the purpose of this study, exclusion criteria were: (1) diagnosis of JIA according to the international league of associations for rheumatology (ILAR) criteria [10], (2) orthopaedic cause of knee complaints, (3) autoimmune disorder other than JIA, (4) reactive arthritis due to an infection (e.g. streptococcal disease, Lyme disease, parvo infections), (5) history of intra-articular corticosteroid injection within the last six months, (6) the need for anaesthesia during the MRI examination, and (7) general contraindications for MRI.

This study was performed in accordance with the Declaration of Helsinki and the local medical ethical regulations. The study was approved by our institutional review board and all participants (and parents if the patients were younger than 16 years old) gave written informed consent.

### Clinical assessment

Physical examination was performed by the same experienced paediatric rheumatologists during the research period. The clinical assessment consisted of a 71-joint count defining the presence of swelling, pain on motion/tenderness and limited range of motion. A physician’s global assessment of disease activity, a patient’s global assessment of well-being and an assessment of patient’s pain were all measured on a 100-mm visual analogue scale (VAS). The Dutch version of the childhood health assessment questionnaire (CHAQ) was used to evaluate functional ability [11, 12]. Laboratory tests included erythrocyte sedimentation rate (ESR) and C-reactive protein (CRP) levels.

According to the ILAR classification, all patients were clinically evaluated and reclassified if necessary. The eventual confirmation or rejection of a diagnosis of JIA was made – by the same paediatric rheumatologist for each patient – after a minimal follow-up period of 6 months [10].

### MRI protocol

MR images were obtained using an open-bore 1.0-T magnet (December 2008–January 2015; Panorama HFO, Philips Medical Systems, Best, The Netherlands) and a 3.0-T magnet (February 2015–July 2016; Ingenia (Omega), Philips Medical Systems, Best, The Netherlands) using a dedicated knee coil. No sedation was used. The children were placed in the supine position with the knee joint centrally in the magnetic field. Contrast-enhanced MRI of the clinically most involved knee (target joint) was performed. If there were no differences in clinical activity between knees, the right knee was considered to be the target joint. For both machines sequences included sagittal T2-weighted fat saturated images, coronal T2-weighted fat-saturated images and axial T2-weighted fat-saturated images before contrast injections, and sagittal T1-weighted images obtained after intravenous contrast injection, and sagittal and axial T1-weighted fat-saturated images obtained after intravenous contrast injection. To provide an optimal discrimination between enhancing synovium and joint effusion, in all patients post-contrast images were obtained in the early phase (< 5 min) after intravenous injection of gadolinium (0.1 mg/kg of body weight, gadobutrol; Schering, Berlin, Germany) [13].

### Image analysis

To prevent any bias, the MRI dataset of included patients was randomly distributed in a larger dataset of MR images of JIA patients. Two experienced readers (with 7 and 19 years of experience, respectively, in musculoskeletal radiology) independently evaluated all images. Agreement on cases, when a difference between readers in scores was ≥ 1, was obtained afterwards in a consensus reading. The MR images were scored in accordance with the Juvenile Arthritis MRI Scoring (JAMRIS) system. This scoring method has been validated and described before in detail [14]. The enhancing synovial membrane was measured at six locations: patellofemoral, suprapatellar recesses, infrapatellar fat pad, adjacent to the anterior and posterior cruciate ligaments, medial posterior-condylar and lateral posterior-condylar. Consequently, the maximal thickness (mm) of any slice at each site was graded as follows: grade 0 if < 2 mm, grade 1 if ≥ 2–4 mm and grade 2 if ≥ 4 mm, resulting in a minimum score of 0 and a maximum score of 12.

Bone marrow changes, cartilage lesions and bone erosions were scored at eight locations: lateral patella, medial patella, medial femur condylar, lateral femur condylar, medial weight-bearing region of the femur, lateral weight-bearing region of the femur, medial tibia plateau and lateral tibia plateau. Bone marrow changes suggestive of bone marrow oedema were scored semi-quantitatively based on the subjectively estimated percentage of involved bone volume at each site as follows: grade 0, none; grade 1, < 10 % of the whole bone volume or of the region of the cartilage surface area; grade 2, ≥ 10–25 % of the whole bone volume or of the region of the cartilage surface area; grade 3, ≥ 25 % of the whole bone volume or of the region of the cartilage surface area, resulting in a minimum score of 0 and a maximum score of 24.

Additionally, joint effusion was evaluated by measuring the diameter of the largest pocket in the knee joint, and scored when the maximal diameter of the largest pocket was ≥ 3 mm as previously described in the International Prophylaxis Study Group (IPSG) scoring method [15]. Consequently, the maximal diameter was graded as follows: grade 0 if ≤ 3 mm, grade 1 if > 3 to ≤ 5 mm, grade 2 if > 5 to ≤ 10 mm, and grade 3 if > 10mm, resulting in a minimum score of 0 and a maximum score of 3.

### Statistics

Descriptive statistics were reported in terms of percentages, means, medians, ranges, interquartile ranges and standard deviations. The independent samples t-test, Fisher’s exact test, Kruskal-Wallis test and the Mann–Whitney *U* test were used to analyse differences between included subgroups. All tests assumed a two-tailed probability, and a p-value of less than 0.05 indicated a statistically significant difference. Data were analysed using SPSS version 24.0 (SPSS, Chicago, IL, USA).

## Results

### Patients

In this study, findings from 57 healthy children (77.2 % female patients) with a mean age of 13.6 years (SD 3.1, range 6.7–17.8 years) were included and analysed.

Of the included children, 31/57 (54.4 %) had passing knee complaints without a known cause, 8/58 (14.0 %) had joint complaints based on a hypermobility syndrome, and 18/58 (31.6 %) of the included children had complaints based on a functional disorder or chronic pain syndrome.

No significant differences in descriptive statistics, clinical findings or laboratory findings were found between the three different subgroups of children included. An overview of the clinical characteristics of the included patients is depicted in Table 1.

**Table 1:** Patient characteristics of the 57 healthy children^a^

	*n* = 57
No. (%) of female patients	44 (77.2)
Age at study visit, mean years (SD)	13.6 (3.1)
Physician’s global assessment of overall disease activity^b^	11 (5–22)
Patient’s global assessment of overall well-being^b^	50 (33–59)
Patient’s pain assessment^b^	59 (40–75)
C-HAQ score^c^	1.000 (0.625–1.500)
Erythrocyte sedimentation rate, mm/h	4 (2–6)
C-reactive protein level, mg/L	1 (0–1)

^a^Except where otherwise indicated, values are median (interquartile range)

^b^Measured on a 0–100 mm visual analogue scale (0 = best, 100 = worst)

^c^Units; 0 = best, 3 = worst

### MRI findings

Of the included children, 40 (70.2 %) were scanned by using the 1.0-T magnet and 17 (29.8 %) children were scanned using the 3.0-T magnet. No significant differences in MRI findings were found between the two systems. Moreover, no significant differences in MRI findings were found between the three different subgroups of children included.

#### Synovial membrane

All of the included patients had a JAMRIS synovial thickness score of 0, meaning that none of the included children had a synovial membrane thickness of ≥ 2 mm.

**Fig. 1:**
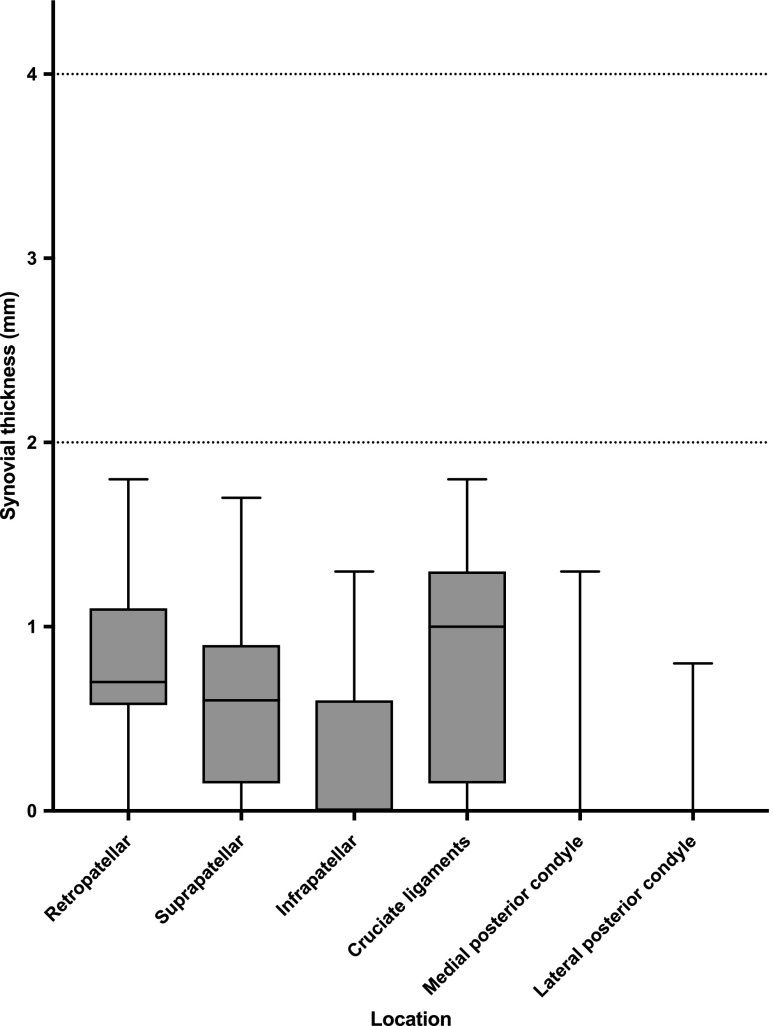
Boxplot (median, interquartile range, min–max) showing the contrast-enhanced thickness of the synovial membrane in 57 healthy children per Juvenile Arthritis MRI Scoring (JAMRIS) location. The horizontal dotted lines indicate the cut-off value of a JAMRIS synovial thickness score of 1 (> 2 mm) and a score of 2 (> 4 mm). As shown, in none of the cases did the synovial thickness exceed the cut-off value of a JAMRIS synovial thickness score of 1

**Fig. 2:**
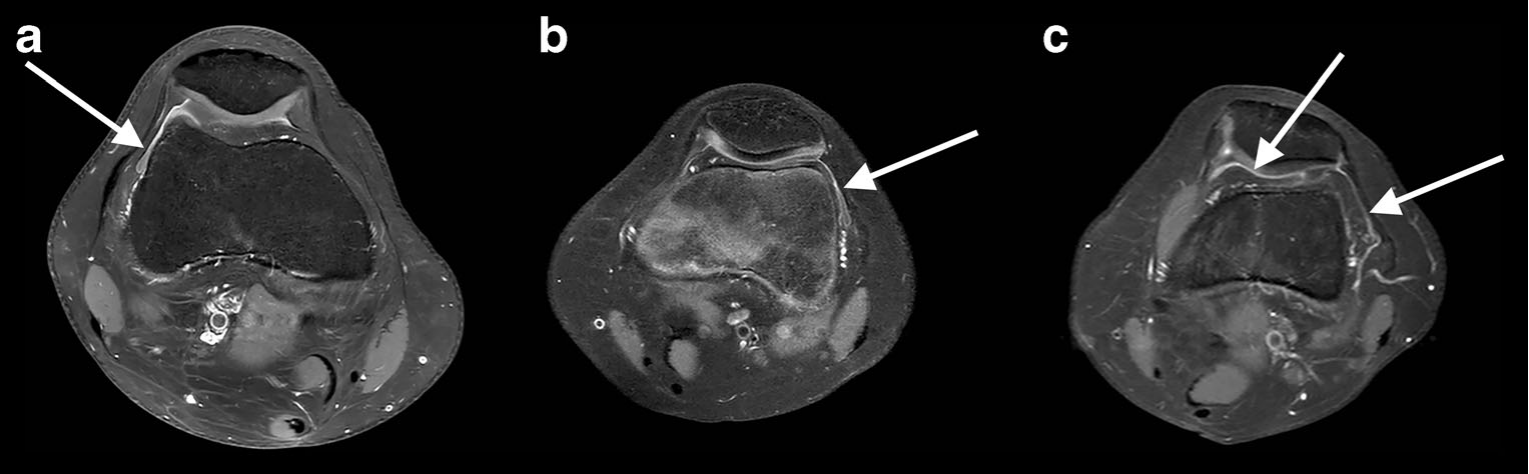
Normal enhancing synovial membrane in three different healthy children (arrows). Axial T1-weighted fat saturated images after the administration of an intravenous contrast agent

As described above, the thickness of the synovial membrane was measured at six predefined anatomical locations, the overall mean thickness of the normal synovial membrane was 0.4 mm (min–max, 0.0–1.8 mm). As shown in Fig. 1, the normal synovial thickness differed slightly within the knee joint, being thickest around the cruciate ligaments and retropatellar and suprapatellar regions. Figure 2 shows some examples of the normal enhancing synovial membrane.

#### Joint fluid

As described above, joint effusion was scored according to the IPSG scoring method. The mean joint effusion score was 0.51 mm (min–max, 0–2). Grade 0 was observed in 29 (50.9 %) patients, grade 1 in 27 (47.4 %) children and grade 2 joint effusion in one (1.8 %) child.

**Fig. 3:**
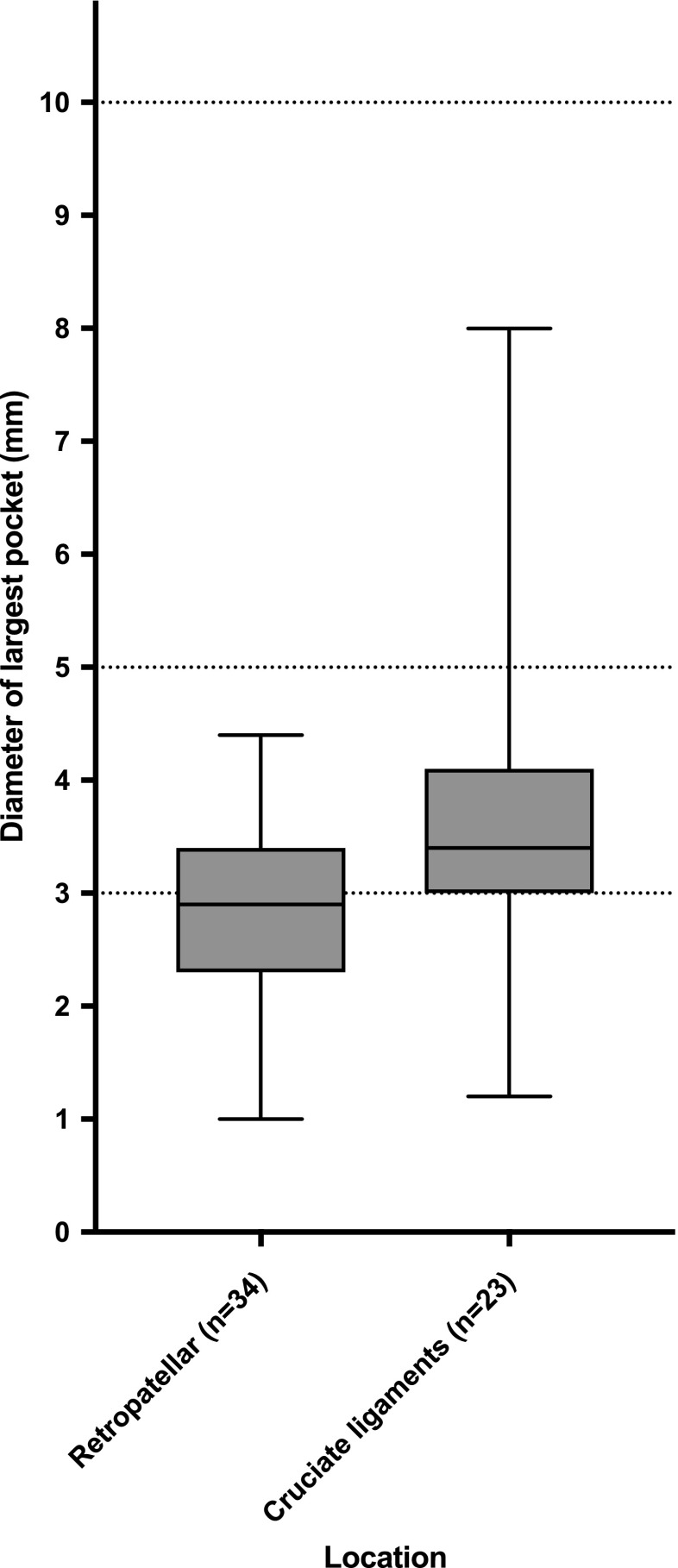
Boxplot (median, interquartile range, min–max) showing the diameters of the largest pocket of joint fluid in 57 healthy children (in 34 children the largest pocket was located at the retropatellar region, whilst in 23 children it was located around the cruciate ligaments). The horizontal dotted lines indicate the cut-off value of International Prophylaxis Study Group (IPSG) joint effusion grades 1 (> 3 mm), grade 2 (> 5 mm), and grade 3 (> 10 mm). As shown, a number of the healthy children scored a grade 1 joint effusion and one child scored a grade 2 joint effusion. In none of the cases was the IPSG joint effusion score grade 3

**Fig. 4:**
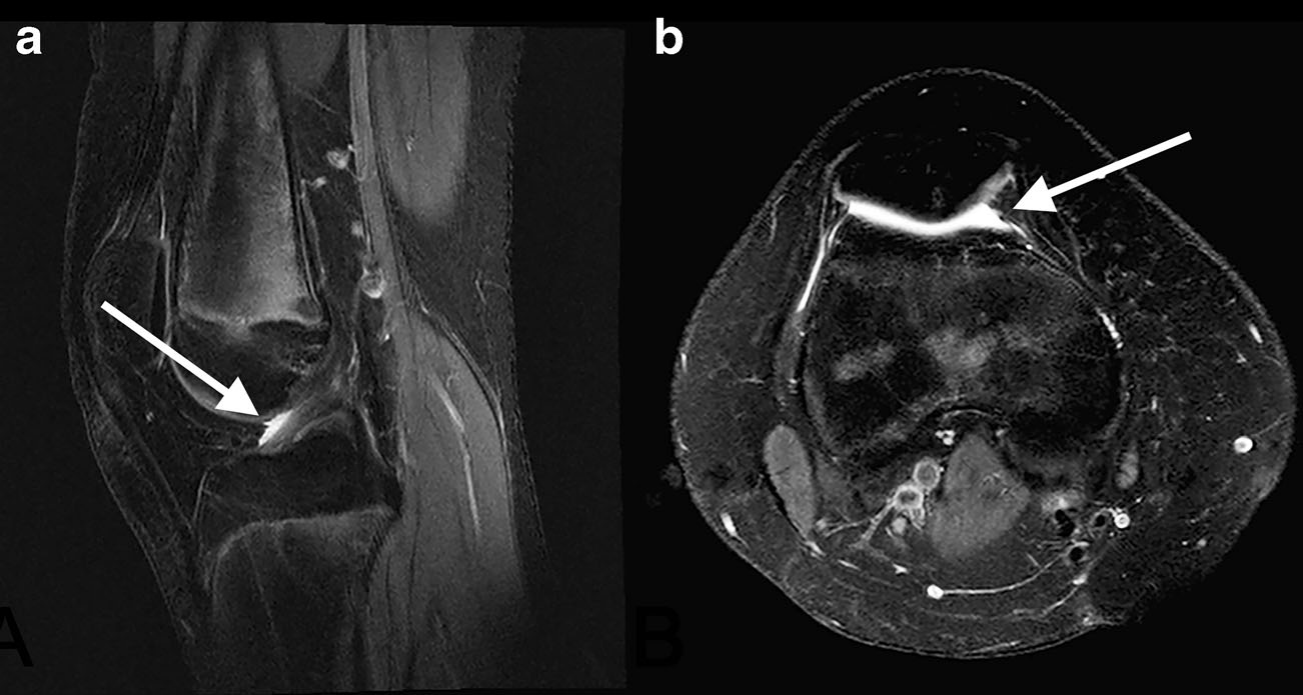
Joint fluid in two different healthy children (arrows). (**A**) Sagittal and (**B**) axial T2-weighted fat-saturated images

The location with the largest pocket of joint fluid was located at the retropatellar region in 34 (59.6 %) children. In 23 (40.4 %) children, the largest pocket was located around the cruciate ligaments. The mean overall diameter of the largest pocket of joint effusion was 2.8 mm (min–max, 0.9–8.0mm). The difference in the diameter of the largest pocket in both regions is depicted in Fig. 3. Some examples of normal joint fluid are shown in Fig. 4.

#### Bone marrow changes suggestive of bone marrow oedema

**Fig. 5:**
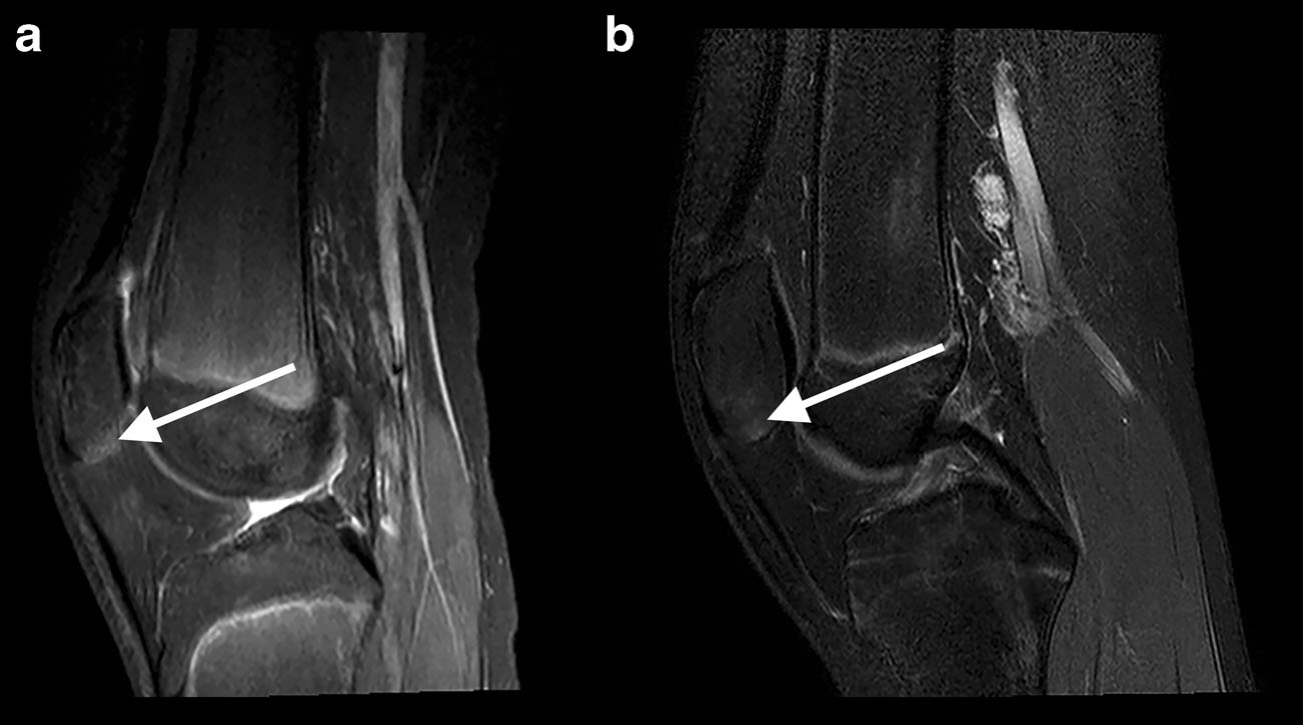
Bone marrow changes in two different healthy children (arrow). Sagittal T2-weighted fat saturated images

Bone marrow changes suggestive of bone marrow oedema were observed in three girls (7.9, 11.7 and 15.9 years of age, respectively). All of these three children had a JAMRIS bone marrow oedema score of 1 located in the apex patellae (see Fig. 5 for an example). As can be seen in Fig. 5, the bone marrow changes had no relation with the patellar tendon or the retropatellar cartilage, and the infrapatellar fat pad had normal signal characteristics in these three children. Moreover, no specific anterior/patellar pain was observed in these three children.

Of these three girls, two were eventually diagnosed with a pain syndrome, whilst one had passing knee complaints without a known cause. No bone marrow changes suggestive of bone marrow oedema were observed in any of the other included healthy children.

#### Cartilage and bone lesions

None of the included healthy children showed cartilage lesions or bone abnormalities suggestive of bone erosions.

## Discussion

In this study, we defined normative standards for the knee of healthy children using contrast-enhanced MRI. We showed that the normal synovial membrane is maximally 1.8 mm thick and that some joint fluid and bone marrow changes suggestive of bone marrow oedema in the apex patellae can be seen in healthy children without arthritis.

Within the past decade, the use of MRI and advances in MRI techniques have substantially improved the evaluation of joint abnormalities in JIA patients [1]. Currently, MRI is considered to be the state-of-the art imaging technique in this respect [2]. Although MRI has previously been shown to be more sensitive than physical examination in the detection of joint inflammation [16, 17], the correct and accurate interpretation of the images is hampered by a lack of normative data for the knee. The lack of normal values complicates the distinction between the normal appearance of joints in children and the pathology present in JIA, as recently also reported for the wrists of healthy children [8, 9].

Since synovitis is the hallmark of disease activity in JIA, it is important to determine the normal appearance and thickness of the synovial membrane on MRI in order to be able to distinguish the normal enhancing synovial membrane from pathological thickened synovium. The current study is the first to determine the normal contrast-enhanced synovial membrane in knees of healthy children, using a standardised imaging protocol with post-contrast images obtained in the early phase (< 5 min) after intravenous injection of gadolinium. Nusman et al. determined the appearance of the synovial membrane on contrast-enhanced MRI of the knee in children with inflammatory bowel disease (IBD) who were clinically unaffected by arthritis [7]. They observed a thickened synovial membrane (> 2 mm) in over 50 % of the included patients, while in none of the children was a synovial thickness of > 4 mm observed. These results are in contrast with our current study. Two important reasons for the observed discrepancies can be pointed out. First of all, our cohort included children who had no (inflammatory) arthritis or a disorder that might result in an associated or accompanying arthritis. This is in contrast to the study performed by Nusman et al. who included – as the best option available at the moment – children with an auto-inflammatory disorder (IBD), in which (subclinical) arthritis may occur. Secondly, in the study performed by Nusman et al., contrast-enhanced images of the knee were scanned a mean of 10.5 min after the intravenous injection of the contrast agent [7]. Recently, Rieter et al. showed that the synovial thickness scores in children with JIA are significantly higher when based on the late post-contrast images (10 min) as compared to the early post-contrast images [13], due to the diffusion of the contrast medium into the joint space over time. Therefore, our current dataset – in which the maximal synovial thickness was < 2 mm in all children – is, in all probability, a more reliable reflection of the true ‘normal appearance’ of the synovial membrane as we included healthy children and post-contrast images were obtained in the early phase.

Previously, we showed that the JAMRIS system is a reliable and responsive outcome measure for the evaluation of disease activity in children with JIA [14, 18]. Our current results indicate that the JAMRIS synovial hypertrophy cut-off value of 2 mm can be considered as a reliable measure, as the maximal thickness of the synovial membrane in the included healthy children was 1.8 mm thick. Thus, we can add another piece of support for the construct validity of the JAMRIS system for the knee.

In our cohort, only 3/57 (5.3 %) of the healthy children showed bone marrow changes suggestive of bone marrow oedema. In these three children, it was located in the apex patellae without other accompanying abnormalities (e.g. abnormal signal of the patella tendon, Hoffa’s fat pad or the overlying cartilage). Moreover, no patellar/anterior knee pain was observed in these three girls, making it unlikely that the observed bone marrow changes are a reflection of pathology but should, therefore, in all probability be considered as a normal variant in growing joints [19, 20].

Moreover, we did not observe any osteochondral abnormalities/variants in the knee that may mimic cartilage lesions or bone erosions. Our results are in contrast with the MRI findings in wrists of a large Norwegian cohort of healthy children [8]. The observed difference can be the result of a more complex and more variable ossification of the carpal bones compared to the ossification that can be seen in the knee.

In the current study, we additionally evaluated the presence of joint fluid. We showed that, according to the IPSG joint effusion score, a score of ≥ 1 was present in 28 (49.1 %) healthy children. Our results might indicate that the IPSG joint effusion score – as it is now – is not very specific for identification of pathological joint effusion in knees of children. For future studies, it would be interesting to evaluate the performance of an adapted IPSG joint effusion score, for instance by leaving out the first grade (> 3 mm) and start grading the amount of joint fluid when the largest pocket is > 5 mm. On the other hand, one should be aware that our contrast-enhanced MRI study – due to medical ethical motives – did not include completely healthy asymptomatic children. Therefore, it would be interesting to evaluate the presence and level of joint fluid in another cohort of completely healthy asymptomatic children by using non-contrast MRI.

Our study has some limitations. Due to ethical issues, it is hard to perform contrast-enhanced MRI studies in a cohort of completely healthy children. Therefore, the included children did have knee complaints at presentation. Although our inclusion and exclusion criteria resulted in a group of children without (inflammatory) arthritis or a disorder that might result in an associated/accompanying arthritis, they were not free of symptoms. Moreover, we included, amongst others, children with a hypermobility syndrome that clinically may mimic JIA [21, 22]. However, for as far as we know, no imaging data are available regarding the presence of synovitis, joint effusion and bone marrow oedema in children with a hypermobility syndrome. Besides, no differences were observed regarding clinical, laboratory and MRI imaging parameters between the included subgroups of children. Nonetheless, our findings might, although not very likely, overestimate the thickness of the synovial membrane and the presence of joint fluid in knees of these ‘healthy children’.

At present, we still need an intravenous contrast agent in order to accurately evaluate the synovial membrane [4], making it very difficult to perform MRI studies in healthy controls. Within the past few years, diffusion-weighted imaging (DWI) has been introduced in JIA as a feasible non-invasive imaging technique for the visualisation of the synovial membrane in the knee joint [23, 24]. This technique might help us in the coming years to evaluate the thickness of the synovial membrane in a cohort of healthy children without any knee complaints whatsoever. Secondly, there might be some inclusion bias since the included children had a mean age of 13.6 years (range 6.7–17.8 years). Therefore, our results cannot easily be translated to younger children. Again, with the use of non-invasive imaging techniques such as DWI, it might be possible to evaluate the normal synovial membrane thickness in future studies in younger children.

In conclusion, we defined normative standards for the knee in healthy children using contrast-enhanced MRI. We showed that the normal synovial membrane proved to be maximally 1.8 mm thick, indicating that the JAMRIS cut-off value of 2 mm can be considered as a reliable measure for evaluating synovial hypertrophy. Moreover, it is important to be aware that some joint fluid and bone marrow changes suggestive of bone marrow oedema in the apex patellae can be seen in healthy children.
